# Cryptococcosis with pulmonary cavitation in an immunocompetent child: a case report and literature review

**DOI:** 10.1186/s12879-024-09061-1

**Published:** 2024-02-06

**Authors:** Qiaoyan Dai, Yingshuo Wang, Qianqian Ying, Qidong Ye

**Affiliations:** 1 Department of Pediatrics, The First Affiliated Hospital of Ningbo University, Ningbo, 315000 China; 2 Zhejiang University School of Medicine, Hangzhou, 310000 China; 3 Department of Pulmonology, Children’s Hospital, Zhejiang University School of Medicine, National Clinical Research Center for Child Health, Hangzhou, 310000 China; 4 Department of pediatrics, Ningbo First Hospital, Ningbo, 315000 China

**Keywords:** Pulmonary cavitation, Pediatric pulmonary cryptococcosis, Immunocompetent, Chest imaging, Case report

## Abstract

**Background:**

Pulmonary cryptococcosis (PC) rarely occurs in immunocompetent children.

**Case presentation:**

A 13-year-old boy was admitted to the First Affiliated Hospital of Ningbo University in February 2023 with complaints of cough and chest pain. Physical examination showed slightly moist rales in the right lung. Chest computed tomography (CT) suggested a lung lesion and cavitation. Blood routine test, lymphocyte subsets, immunoglobulin, and complement tests indicated that the immune system was normal. However, the serum cryptococcal antigen test was positive. Next-generation sequencing revealed *Cryptococcus* infection. The child was diagnosed with PC and was discharged after treating with fluconazole 400 mg. Four months later, chest CT showed that the lung lesion diminished, and reexamination of serum cryptococcal antigen test turned positive.

**Conclusion:**

PC should be considered in an immunocompetent child with pulmonary cavities with nonspecific symptoms.

## Background

Cryptococcosis is a systemic opportunistic fungal infection that occurs frequently in immunocompromised individuals [1]. It is contracted by inhaling a fungus in the *Cryptococcus* genus that can be found in the excrement of birds such as pigeons and parrots. *Cryptococcus* can also be found in fruits and vegetables, as well as on pets, cockroaches, and other animals [2]. There have been very few reports of *Cryptococcus* pneumonia in immunocompetent humans recently [3]. Data on its occurrence in children are even more limited, particularly in developing countries [4]. Therefore, the diagnosis of cryptococcosis in immunocompetent children is challenging. The clinical symptoms of pulmonary cryptococcosis (PC) are nonspecific and frequently manifest as fever, cough, dyspnea, chest pain, and abdominal pain. However, PC can also present as asymptomatic. Thus, cryptococcosis is easily misdiagnosed or ignored. This study presented a case of cryptococcosis with pulmonary cavities in an immunocompetent child.

## Case presentation

**Fig. 1:**
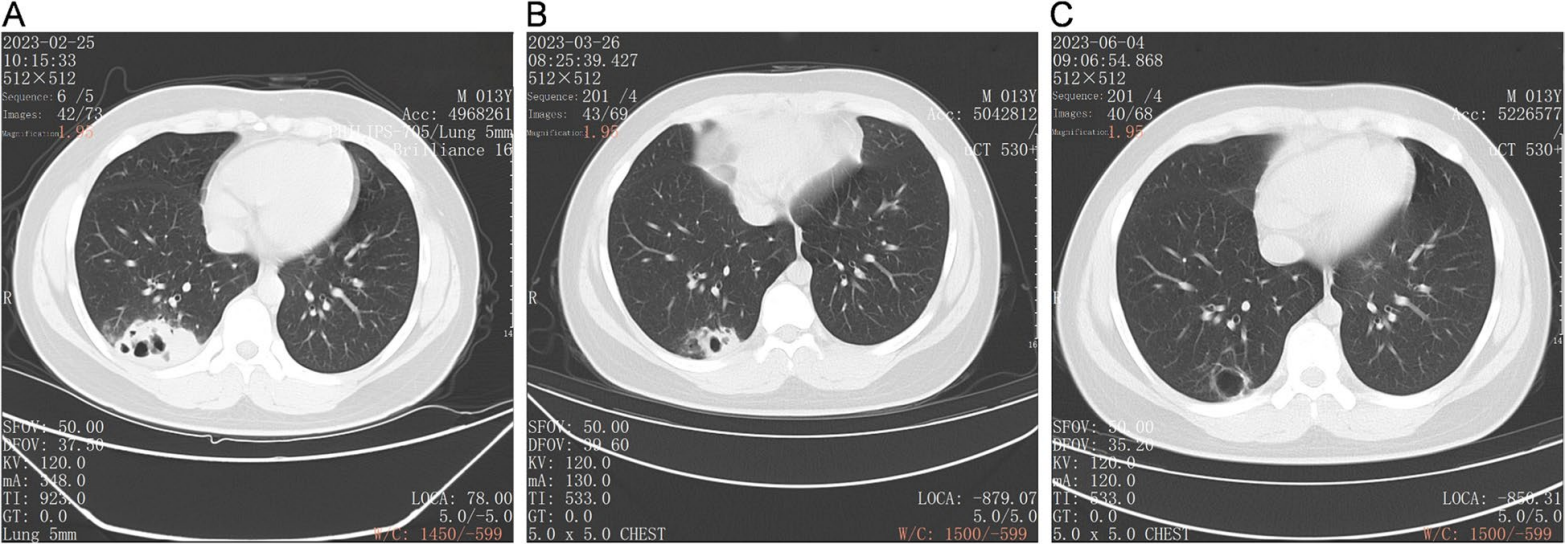
Images. **a** CT on admission showed a lesion and cavities in the right lower lobe of the lung, suggesting infection. **b** CT on the recovery phase (after taking fluconazole orally for 1 month) showed the lesion in the lower lobe of the right lung had reduced. **c** CT on the recovery phase (after taking fluconazole orally for 4 month) showed the inflammation in the lower lobe of the right lung had well absorbed

A 13-year-old boy was admitted to the First Affiliated Hospital of Ningbo University in February 2023 with complaints of cough and chest pain. He had no history of disease or poultry contact. Physical examination showed that he was 173 cm tall and weighed 80 kg, with clear consciousness, a scar of Bacille Calmette-Guerin vaccination, and slightly moist rales in the right lung. There were no abnormalities in routine blood test or blood chemistry. Immunoglobulin and complement tests showed normal IgE levels and lymphocyte subsets. The erythrocyte sedimentation rate was 2 mm/h, and the tuberculosis-SPOT test was negative. The results of the rheumatoid test, as well as the tests for syphilis, hepatitis B, and human immunodeficiency virus (HIV) antibodies, were all negative. In addition, the 13 respiratory pathogen tests also yielded negative results. The aspergillus IgG antibody and galactomannan tests were both negative. However, the serum cryptococcal antigen (CrAg) test, performed by gold immunochromatography assay (Trade name: CrAg Lateral Flow Assay; Product code: CR2003; Manufacturer: Immuno-Mycologics, Inc) was positive 2 days after admission. Both routine and biochemical test of cerebrospinal fluid (CSF) showed normal results, and india ink staining for *Cryptococcus* was negative. Blood culture, CSF culture, and sputum culture were all negative. Cardiac ultrasound, electrocardiogram, cerebral magnetic resonance imaging, and fiber bronchoscopy revealed no significant abnormalities. A chest ultrasound revealed heterogeneous echoes in the right thoracic cavity with possible inflammatory wrapping. The chest computed tomography (CT) demonstrated a lesion and cavities in the right lower lobe of the lung (Fig. 1a). Bronchoscopy showed that the cavity was unobstructed. In light of the unexplained pulmonary cavitation, absence of abnormalities in the appearance of the tracheal structure, and a positive blood CrAg test, alveolar lavage fluid was subjected to next-generation sequencing (NGS) for a definitive diagnosis. This was done to exclude false positives and determine the underlying cause of the disease. BALF samples with a total cell concentration of ≥1 × 10^6^ cells/mL underwent pre-processing for host removal using sample release reagent (Genskey Co., Ltd., Beijing, China). Subsequently, DNA was extracted from 0.3 mL BALF samples following the instructions of the DNA Extraction and Purification Kit (Genskey Co., Ltd., Beijing, China). The DNA library was generated after cDNA synthesis, and its quality was assessed through Qubit detection. Finally, the qualified library was sequenced on an MGISEQ200 platform (MGI Tech Co., Ltd., Shenzhen, China). Adapters and low-quality reads (Q, 20) were removed from raw data with 50-bp single-end reads using fastp software. Human sequence data were excluded by mapping onto the human reference database (hg38, YH genome, T2T-CHM13 genome) through Burrows Wheeler Aligner (BWA) software. The remaining sequence reads, aligned to the Dian Diagnostics Pathogenic Microorganism Genome Database by BWA, underwent annotation and statistical analysis. Negative controls (Genskey Co., Ltd., Beijing, China) underwent the same sequencing and bioinformatics analysis procedures as the clinical samples. Synthesized sequences were used as a positive control, serving as indicators for the detection process in each batch. For homogenized species-specific sequences, the prevalence of microbiota was determined according to the mNGS criteria. For clinical core pathogens and bacteria difficult to detect, including firmicutes and intracellular bacteria, reads ≥1 were considered positive. For other clinically relevant bacteria, fungi, and viruses, reads ≥3, exceeding those in the negative controls of the same batch, were considered positive. For bacteria, fungi, and viruses not clinically reported or isolated, reads ≥20, surpassing those in the negative controls of the same batch, were considered positive. The NGS test detected *Cryptococcus* species in the bronchoalveolar lavage fluid at 3 days after admission (Table 1). As the cryptococcal infection was confined to the lungs, PC was the definitive diagnosis. He was treated with intravenous injection of fluconazole at 400 mg once a day for 8 days, followed by continuous oral administration of fluconazole capsules at 400 mg once a day for 6 months. The child’s symptoms, including chest pain, were significantly relieved. He was discharged 10 days after admission without coughing or chest pain. Chest CT revealed that the lesion in the lower lobe of the right lung reduced after taking fluconazole for 1 month (Fig. 1b). The lesion in lung diminished and reexamination of serum CrAg turned positive after taking fluconazole for 4 months (Fig. 1c).

**Table 1:** Next-generation sequencing of the bronchoalveolar lavage fluid

Generic			Specific		
Generic name	Relative abundance	Sequence number	specific name	confidence coefficient	Sequence number
*Cryptococcus*	66.10%	22	*Cryptococcus neoformans*	99%	19

## Discussion

This case presented an immunocompetent child with PC manifested by pulmonary cavities, who were diagnosed by NGS and cured with fluconazole.

PC is a fungal lung disease caused by the inhalation of *Cryptococcus* spores. It can occur in both immunocompromised individuals and immunocompetent hosts, but T-cell deficiency confers the most susceptibility [5]. The diagnosis of PC is challenging, and it is often misdiagnosed as other diseases, including pneumonia, lung abscess, tuberculosis, or lung malignant tumor [6]. Previous studies reported consistent radiological manifestations of PC in children compared with those in adults, with localized nodular lesions being the most frequent radiographic finding in the chest [7]. There are several reports of PC in immunocompetent children [2, 4, 8, 9]. PC can present as an intrapulmonary cavity with an incidence of 11.0–34.6% [10–12] as this case presented. A similar case of a Chinese 16-year-old girl reported bilateral diffuse cavity nodules [8]. The common chest imaging of PC includes nodule, masses, consolidation, and pleural effusion [13–15]. In addition, abnormal imaging findings of cryptococcosis, such as nodules, pulmonary infiltration, hilar lymph node enlargement, and pulmonary cavity, were also found in tuberculosis [16]. It is important that health professionals become familiar with these diseases since they are becoming more important around the world as a result of travel and immigration [17] (Table 2). Previous case reports of PC in immunocompetent children are scarce, but boys aged between 5 and 17 years seemed more affected [2, 4, 18], which was consistent with this case.

**Table 2:** Literature review of PC cases

Author	Year	Clinical presentation	Diagnostic methods	Risk factors	Diagnosis	Outcome	Critical findings
Bauer et al. [2]	2010	16/M, No clinical symptoms, abnormal X-ray	1.CT: multiple nodules in the left inferior lobe of the lung 2. Fine needle aspiration biopsy	A history of close contact with pigeons	Cryptococcal pneumonia	Oral fluconazole for 8 weeks with regression	Isolated PC in an immunocompetent boy
Haider et al. [3]	2022	69/M, Cough, shortness of breath, fatigue for 3 months	1. CT: extensive pneumonic infiltrates in the left lower lobe. 2. Fungal smear and culture from the bronchial brushing	Smoking history	PC combined with lung adenocarcinoma	6 weeks of oral fluconazole with improved lesion, and chemotherapy for lung adenocarcinoma was started.	Cryptococcal pneumonia in an immunocompetent old male
Nakatudde et al. [4]	2021	8/M, Productive cough, low-grade fevers, night sweats, weight loss, and on and off right-sided chest pain for 1 year	Fine needle aspiration biopsy confirmed the diagnosis of PC and serum cryptococcal antigen test was positive.	No	PC	The child improved on amphotericin and fluconazole treatment.	PC in an immunocompetent boy in Uganda
Huang et al. [8]	2023	16/F, Dry cough and fever for 10 days	Blood, bone marrow and cerebrospinal fluid cultures were all positive for Cryptococcus	Immunocompromised (history of yolk sac tumor, long-term ITP hormone therapy)	PC, cryptococcal meningitis, cryptococcal osteomyelitis and cryptococcal septicemia	cured by antifungal therapy (amphotericin, fluconazole)	Disseminated cryptococcal infection with pulmonary involvement presenting as diffuse cavitary nodules in an immunocompromised girl
Ismail et al. [20]	2018	5/F, Intermittent fever, dry cough and progressive abdominal distension for 2 months	1. Chest CT: miliary pulmonary shadow 2. Cryptococcus was found in cerebrospinal fluid and blood culture.	No	disseminated cryptococcosis	die	Disseminated cryptococcosis presenting as miliary lung shadows in an immunocompetent girl
Adsul et al. [19]	2019	45/F, Severe back pain for 5 months	Histopathological examination and microbial culture of thoracic vertebrae	1. Pigeons were kept as pets at home 2. Diabetes	Cryptococcotic osteomyelitis of the thoracic vertebra with fungal retinal deposits	Recovery of motor function in both lower limbs, but loss of unilateral vision (perception of light only)	Thoracic cryptococcal osteomyelitis mimicking tuberculosis in a female adult
Isaac et al. [29]	2023	62/M, Fever, difficulty short of breath	Blood cryptococcus antigen + antibody test	1. Long-term use of hormones in the context of pulmonary fibrosis after COVID-19 2. Diabetes	Pulmonary cryptococcosis and pulmonary fibrosis	Clinical and imaging tests were significantly improved	Pulmonary cryptococcosis and pulmonary fibrosis as a complication of COVID-19 pneumonia in an old male
Zhang et al. [9]	2021	3/M, 7/M, 10/F; Fever, dry cough	1. Lymph node biopsy 2. Cryptococcal antigen test 3. Imaging examination: enlargement of mediastinal lymph nodes	Two patients had a history of pigeon contact. The third patient had no risk factors	PC	All cured	Pediatric pulmonary cryptococcosis cases with prominent manifestation of mediastinal lymphadenopathy in children
Chen et al. [37]	2019	7/F, Cough and recurrent fever for more than 1 month; Bilateral cervical, axillary and inguinal lymph node enlargement; Chest CT: Multiple nodular high-density images in both lungs	Inguinal lymph node biopsy: epithelioid granulomatous lymphadenitis with granulomatous central necrosis; GMS and PAS staining showed cryptococcus	No	Disseminated cryptococcosis is associated with multiple lymph node enlargement and lung involvement	Symptoms disappear; Lymph node enlargement was significantly relieved	Disseminated cryptococcosis with multiple and mediastinal lymph node enlargement and lung involvement in an immunocompetent girl.
Yao et al. [38]	2020	72/M, Repeated dry cough for over half a year; Chest CT revealed an irregular mass in the hilum of the left lung and two small nodules in the right lung.	Percutaneous lung biopsy, bronchofiberscopy and surgical pathology results	No	Pulmonary cryptococcosis coexisting with central lung cancer	Pulmonary cryptococcal nodules disappeared	Pulmonary cryptococcosis coexisting with central type lung cancer in an immuocompetent old male

The clinical symptoms of PC may be similar to those of tuberculosis, leading to misdiagnosis [19], particularly in areas with high incidences of tuberculosis, such as Asia and Africa [4]. In most cases, delayed diagnosis leads to death [20]. It has been reported that five of the 11 patients with cryptococcosis who were misdiagnosed as having tuberculosis died due to delayed diagnosis and the start of antifungal therapy [21]. This was the first time NGS was employed for the diagnosis of PC. Typically, PC is diagnosed through histological results, fungal cultures, serum CrAg, and imaging [22]. However, in this particular case, NGS, rarely utilized in previous cases, played a crucial role in achieving an early and definitive diagnosis of PC. This contribution proved instrumental in improving the overall outcome.

In this study, the patient presented with respiratory symptoms such as cough and chest pain. In addition, if not detected and treated promptly, cryptococcosis can result in life-threatening disseminated infection and respiratory failure [3]. According to statistics, annually there are 220,000 cases of cryptococcal meningitis in immunocompromised individuals, leading to nearly 180,000 deaths [23]. The incidence of PC increased by more than six times within several years, reaching 38 cases per million in 2006, among whom most of the increased cases are HIV-negative [24]. A study in Uganda reported 11% of patients with human immunodeficiency virus (HIV) infection developed PC [25]. In mainland China, 15.7% of the cryptococcosis patients were AIDS or HIV-infected [26], while 60% of PC cases were diagnosed in immunocompetent non-HIV patients [27]. A recent study reported the most common types of *Cryptococcus* infections in Southern China were cryptococcal meningitis, cryptococcal fungemia, and PC, and only 12 out of the 170 patients had autoimmune disorders [28]. According to the local Health Department in the Ningbo city, there were 9 cases of adult with PC over the past year, all with normal immune function, and the present case was the only case of PC in child. Additionally, the COVID-19 pandemic may increase the number of patients at risk [29], and PC was diagnosed 1 month after a novel coronavirus infection.

Cryptococcosis is usually diagnosed by microscope, immunology, or microbiology [30]. Direct detection of ink staining is a rapid, low-cost method for detecting *Cryptococcus neoformans*. Although the specificity is high, the sensitivity about 86% depends on the user, and it is inadequate for detecting early fungal infection [31]. In recent years, CrAg test, a rapid and sensitive diagnostic method, has received increasing attention as a novel diagnostic tool for PC [32] and recommended by guideline [33]. However, in some cases of non-disseminated PC, CrAg may not be detected despite the presence of infection [7]. In this case, the infection was limited to the lungs, but a positive CrAg test was detected. Therefore, CrAg testing cannot be ignored in PC patients, and a negative result should not necessarily rule out cryptococcal infection. Although PC in immunocompetent adults may resolve on its own, children have relatively underdeveloped immunity, which increases the risk of dissemination. Consequently, active antifungal therapy is recommended for pediatric PC. Fluconazole has high bioavailability and fewer side effects. Therefore, it is recommended as the first-line treatment for PC in immunocompetent children [9]. In this case, the blood cryptococcal antigen was still positive in the outpatient follow-up up to 4 months. Several studies suggested that it took more than 1 year for the blood CrAg to turn negative in the adult department [32].

*C. neoformans var. grubii* serotype A represented over 80% of all *Cryptococcus* globally [34]. However, East Asian countries reported more cryptococcal polymorphisms than Southeast Asian countries [35, 36].

In conclusion, a diagnosis of PC should be considered in immunocompetent children present with pulmonary cavities and nonspecific symptoms. NGS test and early treatment of fluconazole would be helpful to a better prognosis.

## Data Availability

All data generated or analyzed during this study are included in this article.

## Abbreviations

PC: Pulmonary cryptococcosis
NGS: Next-generation sequencing
HIV: Human immunodeficiency virus
CrAg: Cryptococcal antigen
CT: Computed tomography
